# Widespread evidence of viral miRNAs targeting host pathways

**DOI:** 10.1186/1471-2105-14-S2-S3

**Published:** 2013-01-21

**Authors:** Joseph W Carl, Joanne Trgovcich, Sridhar Hannenhalli

**Affiliations:** 1 Center for Bioinformatics and Computational Biology, University of Maryland, College Park, MD, USA; 2 Department of Surgery, The Ohio State University, Columbus, OH, USA

## Abstract

**Background:**

MicroRNAs (miRNA) are regulatory genes that target and repress other RNA molecules via sequence-specific binding. Several biological processes are regulated across many organisms by evolutionarily conserved miRNAs. Plants and invertebrates employ their miRNA in defense against viruses by targeting and degrading viral products. Viruses also encode miRNAs and there is evidence to suggest that virus-encoded miRNAs target specific host genes and pathways that may be beneficial for their infectivity and/or proliferation. However, it is not clear whether there are general patterns underlying cellular targets of viral miRNAs.

**Results:**

Here we show that for several of the 135 known viral miRNAs in human viruses, the human genes targeted by the viral miRNA are enriched for specific host pathways whose targeting is likely beneficial to the virus. Given that viral miRNAs continue to be discovered as technologies evolve, we extended the investigation to 6809 putative miRNAs encoded by 23 human viruses. Our analysis further suggests that human viruses have evolved their miRNA repertoire to target specific human pathways, such as cell growth, axon guidance, and cell differentiation. Interestingly, many of the same pathways are also targeted in mice by miRNAs encoded by murine viruses. Furthermore, Human Cytomegalovirus (CMV) miRNAs that target specific human pathways exhibit increased conservation across CMV strains.

**Conclusions:**

Overall, our results suggest that viruses may have evolved their miRNA repertoire to target specific host pathways as a means for their survival.

## Introduction

MicroRNAs (miRNA) are ~22nt non-coding regulatory genes that target other RNA molecules via sequence-specific hybridization, which results either in translation inhibition (an imperfect target miRNA sequence match) or in cleavage and degradation of the targeted RNA (a perfect target miRNA sequence match) [1]. Initially discovered in worms, miRNAs are now known to serve numerous critical regulatory functions in a wide spectrum of species including viruses [2], plants, and mammals [3]. In mammals, miRNAs play a regulatory role in processes as diverse as metabolism, immune response, cell death, proliferation, circadian rhythm, and hematopoiesis [4]

**Figure 1:**
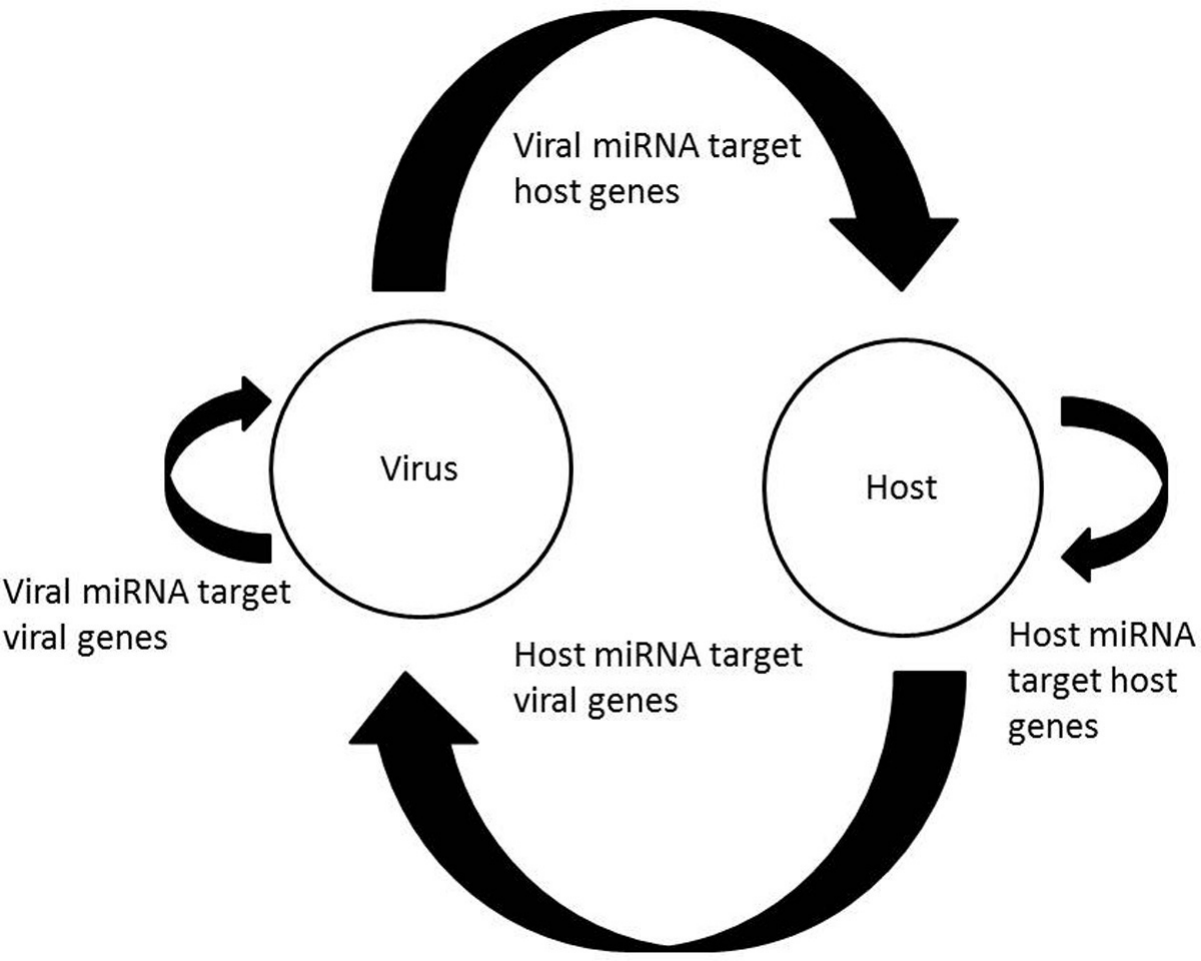
**Possible interactions between miRNA and target genes. (a) Endogenous host miRNAs target specific host genes. (b) Host miRNAs target viral genes. (c) Virus encoded miRNAs target viral genes to mediate immune evasion or maintenance of latency. (d) Viral miRNAs have been shown to target specific host genes**.

Figure 1 depicts all possible miRNA-mediated interactions between a virus and the host. A wide variety of species utilize their miRNAs to target and regulate endogenous pathways (Figure 1a) [5]. Plants, invertebrates[6], and vertebrates [7] utilize their miRNAs to target offending virus (Figure 1b). There are known instances of viruses using their endogenous miRNAs to target their own genes to evade host immune system's surveillance and maintain latency (Figure 1c) [8]. Finally, there are several known instances of viral miRNAs targeting host genes (Figure 1d). For instance, Epstein-Barr virus (EBV) encoded miR-BART5 targets the p53-regulated pro-apoptotic gene PUMA [9]. More recently, EBV miRNAs were determined to predominantly target cellular transcripts during latent infection; 531 sites were identified in 3492 cellular 3' UTRs that contained seed matches to viral miRNAs; of them, 24 were experimentally confirmed [10]. More importantly, these target genes were enriched for cellular processes that facilitated the viral infection. This study suggested that the number of viral miRNA targets in the host may be much greater than previously assumed and are specific to host pathways.

Viruses depend upon the molecular machinery of the cells they infect. They have co-evolved with their host over millions of years and have had to adapt to the cellular environment, which in turn is evolving to evade viral infection. RNA viruses are limited in genomic size due in part to error prone RNA polymerases. DNA viruses allow a larger genomic size and a richer gene repertoire to interact with the host and presumably evolve complex mechanisms for infectivity and survival; we have therefore restricted our study to DNA viruses. DNA viruses have utilized several mechanisms to evade host defenses [11]. Poxviruses and herpes viruses have evolved multiple strategies to disrupt presentation of viral antigens by MHC molecules in order to evade control by T lymphocytes [12]. The adenovirus inhibits MHC-I molecules to evade immune detection [13]. Viruses have also developed mechanisms to bolster the host cell to withstand the viral replication process such as an inhibition of apoptosis [14], an induction of cellular growth or differentiation [15], a recruitment of tissue repair processes such as fibrosis [16], tissue growth [17], and angiogenesis [18].

Evidence presented thus far suggests that viral miRNAs target host pathways and biological processes that may potentially benefit viral replication or persistence. If the suppression of a host gene disables host defense or enables viral survival or proliferation (e.g., by promoting cell growth, or inducing repair mechanisms to aid the survival of the infected cell), then encoding a miRNA that targets the specific host gene would potentially benefit the virus. While a few previous studies support this suggestion with specific examples in a limited context [10,19,20], it is not clear whether there are shared patterns among different viral miRNAs targeting different hosts. Here we investigate the general patterns of cellular targeting by viral miRNAs for a comprehensive set of human and murine viruses.

We found that for several of the 135 known miRNAs encoded by human viruses, the putative human gene targets of these viral miRNAs are enriched for specific host pathways whose targeting is arguably beneficial to the virus. Many of these pathways, including cancer-related pathways, melanogenesis, axon guidance, ErbB, GnRH, Wnt and MAPK signaling pathways are independently enriched among targets of multiple miRNAs encoded by multiple viruses. Next, to assess the generality of our findings for known miRNAs, based on previously identified 6809 putative miRNAs encoded by 23 human viruses, we predicted high-confidence targets of the miRNAs. We then assessed the enrichment of pathways and Gene Ontology (GO) functional classes in the target sets. At a false discovery rate (FDR) of 10%, we found that 75 miRNAs, corresponding to 15 viruses, target 43 unique pathways. Of these pathways, 20 (6 of which are cancer-related) were repeatedly targeted by independent miRNAs encoded by 14 different viruses. A similar analysis of 4 murine viruses encoding 21 miRNAs also yielded several enriched pathways and functions which significantly overlapped with those identified in human. Based on our biological understanding, the most significant pathways and processes likely targeted by viral miRNAs present a reasonable choice from a virus' vantage point, which include cell growth, axon guidance, and antigen processing and presentation. We discuss this in light of current understanding of viral infection pathways. Moreover, based on genome sequences of 14 strains of human CMV, we found that the miRNAs targeting these specific pathways are evolutionarily more conserved than the other viral miRNAs, suggesting a purifying selection of targeting specific host pathways. Overall, our results suggest that viruses may have evolved their miRNA repertoire to target specific host pathways as a means for their survival and proliferation.

## Results

### Targets of experimentally validated Human viral miRNAs are enriched for several pathways and functions potentially important for viral infectivity and survival

There are 135 experimentally identified miRNAs for the human viruses included in our study, which are cataloged on miRBase (http://www.mirbase.org). For each of the 135 known miRNA, we predicted gene targets using the Miranda3.3 tool [21] in the 3' UTR sequences of 3504 human protein coding genes, which had unique ENTREZ IDs in KEGG [22]. We only considered targets with a Miranda score of at least 140 (which corresponds to a perfect heptamer match in positions 2-8). Beyond this baseline threshold, we scrambled each of the 3' UTR sequence and predicted targets in the scrambled sequences following an identical procedure and identified a score threshold at which 1% of the scrambled sequences had a target predicted. For the putative targets of each miRNA, we then identified KEGG pathways (http://www.kegg.jp) enriched in these target gene sets at 1% FDR (see Methods). Table 1 shows the pathways that were enriched in more than one virus, and in more than 3 independent miRNAs. As we discuss in detail later, many of these pathways - cancer, Axon guidance, ErbB, MAPK, and Wnt signaling, etc., are arguably beneficial targets for the virus.

**Table 1:** Targets of known human viral miRNA are enriched for KEGG Pathways.

KEGG Pathway Name	Total (135)	HSV1 (25)	HSV2 (24)	EBV (44)	CMV (17)	KSHV (25)
Cancer	10(27)	2(3)	2(4)	2 (3)	1(6)	3(11)
ErbB signaling pathway	9	3	2	4	0	0
Axon guidance	7	3	1	1	0	2
Melanogenesis	6	2	2	0	1	1
MAPK signaling pathway	5	0	1	0	1	3
GnRH signaling pathway	4	0	0	0	1	3
Wnt signaling pathway	4	1	0	1	1	1

Individual viral miRNAs target human genes that are enriched for KEGG pathways, and multiple viruses target the same pathways even across virus families. We list the pathways that were represented multiple times with a FDR of 1%. The name of the virus and number of known miRNA used for analysis is supplied as the header; the values indicate the number of miRNA that are enriched for the pathway listed. Cancer pathways were aggregated into one category and the value indicates the number of miRNA that are enriched for a cancer-related pathway and in parenthesis is the total number of cancer pathways enriched.

### Pathways targeted by known miRNAs are also enriched among targets of predicted viral miRNAs

Given that experimentally identified viral miRNAs represent a potentially very small fraction of all functional viral miRNAs there is merit to exploring high-confidence predicted viral miRNAs for evidence of targeting specific host pathways. We obtained predicted miRNA candidate hairpins for double stranded DNA (dsDNA) viruses from VirMir Database [23]. This includes 6809 miRNA for 23 human dsDNA viruses and 2188 miRNAs for 4 mouse dsDNA viruses (Table 2). We excluded the papillomaviridae family as it contained fewer than 10 predicted miRNAs. For each of the candidate miRNA, we then predicted gene targets as for the experimentally identified miRNAs described above. However, we used a miRNA-specific score threshold at which 5% of the scrambled sequences had a target predicted; this threshold was always much more stringent than the baseline threshold of 140.

**Table 2:** Viral miRNA selection.

Virus	miRNA	Virus	miRNA	Virus	miRNA
Human adenovirus A	20	Human adenovirus Type 35	54	HSV6B	107
Human adenovirus C	104	Human adenovirus Type 5	114	HSV7	54
Human adenovirus D	136	Human adenovirus Type 7	78	HHV8(KSHV)	392
Human adenovirus E	132	HSV1	939	Orf virus	953
Human adenovirus F	58	HSV2	840	Vaccinia virus	38
Human adenovirus Type 11	52	HSV3 (dumas)	111	Muridherpes 1	1158
Human adenovirus Type 1	122	HHV4(EBV)	741	Muridherpes 2	894
Human adenovirus Type 17	136	HHV5(CMV)	1444	Muridherpes 4	98
Human adenovirus Type 2	124	HSV6	98	Murine adenovirus A	38

Species specific viruses containing 10 or more miRNA were selected from VirMir DB. The virus name and number of miRNAa re listed.

Next, as above, we tested whether the predicted viral miRNA targets are enriched for specific KEGG pathways. To accommodate for potentially high false positives in predicted miRNAs, we employed additional criteria for the selection of enriched pathways. After assessing the overlap between the predicted miRNA targets and the genes in the pathway using a hypergeometric test, we further adjusted p-values based on a randomized sampling approach in a pathway-specific fashion, controlling for the lengths of the target genes 3' UTR. Finally, we corrected the adjusted p-values for multiple testing (see Methods). Based on FDR threshold of 10%, a total of 43 unique pathways were enriched for 75 miRNAs in 14 human viruses. Table 3 shows KEGG pathways that were enriched among the targets of miRNAs in more than two viruses.

**Table 3:** Human miRNA targets are enriched for KEGG Pathways and GO terms, and multiple viruses target the same pathways even across virus families.

KEGG Pathway	adeno/herpes/pox		Gene Ontology	adeno/herpes/orf	
			
	#miRNA	# virus		#miRNA	# virus
Biosynthesis of steroids	2/1/0	2/1/0	Cytoplasmic microtubule	0/9/0	0/3/0
Glycerolipid metabolism	0/3/1	0/3/1	Microtubule binding	0/9/0	0/2/0
ErbB signaling pathway	7/0/0	4/0/0	Kinase binding	0/8/0	0/2/0
Regulation of autophagy	2/1/0	2/1/0	Cytoplasmic vesicle membrane	4/4/0	4/3/0
Hedgehog signaling pathway	0/3/0	0/2/0	Inner ear morphogenesis	0/8/0	0/2/0
Axon guidance	3/5/0	2/3/0	Multicellular organismal development	0/7/0	0/5/0
Antigen processing and presentation	3/6/3	2/4/1	Transferrin transport	4/3/0	4/2/0
Long-term potentiation	0/4/0	0/2/0	Protein autophosphorylation	5/2/0	5/1/0
GnRH signaling pathway	2/2/0	2/2/0	Protein serine/threonine kinase activity	0/5/1	0/5/1
Cancer	3/17/7	2/6/1	Extracell-glutamate-gated ion channel activity	5/1/0	4/1/0
			
			Voltage-gated ion channel activity	1/5/0	1/3/0
			Synaptic transmission	1/5/0	1/4/0
			Histone methylation	0/4/2	0/3/1
			Canonical Wnt Receptor signaling pathway	0/4/2	0/3/1

We list the pathways that were represented multiple times with an FDR of 10%. The numbers separated by a slash correspond to adeno, herpes and orf families of viruses.

We assessed whether the distribution of the number of miRNAs targeting a pathway is skewed relative to expectation. Given the bipartite graph composed of miRNAs (whose targets are enriched for specific pathways) and the corresponding enriched pathways, we randomized the bipartite graph while preserving the degree of each miRNA, in order to estimate the expected degree distribution of pathways. The degree distribution for the real data is skewed towards higher values (Kolmogorov Smirnov (KS) test p-value = 0.029). The antigen processing and presentation pathway was specifically enriched among the targets of twelve miRNA. Based on 1000 randomizations of the bipartite graph; the probability of this occurrence is less than 0.0001. Three pathways were enriched in targets of 7 or more miRNAs (p-value = 0.016 based on 1000 randomizations).

### Human viral miRNA targets are enriched for specific GO functions

While the pathway-enrichment analysis can reveal very specific biology, it is also limited by our knowledge of pathways. We therefore extended our pathway-enrichment procedure above to search for enriched GO biological processes. As before, we applied a term-specific adjustment of p-value, applied multiple testing corrections, and used a FDR threshold of 10% to select for enriched GO terms. Table 3 shows GO terms that were enriched among the targets of miRNAs in more than one virus. An interesting set of terms not revealed by KEGG pathway analysis became apparent (discussed later). As above, we analyzed the degree distribution associated with GO terms in the bipartite graph composed of enriched GO terms and miRNAs. As before, the GO terms were multiply targeted more frequently than expected (KS test p-value = 0.007). Cytoplasmic microtubule and microtubule bindings were enriched for 9 miRNA.

It is possible that a pathway/GO term is enriched among targets of multiple miRNAs simply due to a high similarity of the miRNA sequences, and could not be interpreted as independent evidence. To rule out this possibility we analyzed the pair-wise overlap in target sites for miRNAa enriched the same functional term (see Methods). For each miRNA pair, we calculated the overlap as the fraction of all target sites (for the miRNA with fewer hits) that overlapped with the target sites of the other miRNA. We found that in almost all cases (99%), the overlap was less than 0.05. Thus, the miRNA sequence similarity does not explain the common pathway enrichment. Taken together, our results suggest that several pathways and GO terms are targeted independently by multiple miRNAs.

### Human and mouse viruses target similar processes

Next, we investigated whether our finding in human - viral miRNAs tend to target critical host pathways - also holds in other species, and whether similar pathways and functions are targeted in evolutionarily distant species. We used herpes and adeno virus specific for mice and performed an identical KEGG pathway and GO term enrichment analyses as was used for human miRNAs. By necessity, the enrichment was restricted to genes with mapped unambiguous human-mouse orthologs; this reduced the size of the KEGG gene list for enrichment purposes from 3504 genes to 1381 genes, and the pathway list from 201 down to 189. None of the miRNA target sets from the mouse adeno family were enriched for a functional term at the 10% FDR threshold. For the herpes family, we found substantial overlap among the processes and pathways targeted by mouse and human viruses in their respective hosts, namely the ErbB signaling pathway, axon guidance, neuron development, regulation of immune response, and Insulin receptor regulation. Table 4 shows the pathways that were multiply targeted in at least one species. There was only one experimentally verified mouse miRNA included in our study and it was enriched for cell communication pathway, neuron differentiation, and cellular response to retinoic acid processes.

**Table 4:** Mouse and human virus target similar KEGG pathways and GO terms.

KEGG Pathway	Mouse/Human
Synthesis and degradation of ketone bodies	2/1
C21-steroid hormone metabolism	1/2
ErbB signaling pathway	2/4
Axon guidance	2/3
Olfactory transduction	2/2
Cancer	2/5

**GO term**	**Mouse/Human**

Multicellular organismal development	3/5
Neuron development	3/4
Regulation of immune response	4/3
Peptidyl-tyrosine phosphorylation	3/3
Cellular response to retinoic acid	3/3
Integrin binding	3/2
Intermediate filament	3/2
Phosphorylation	3/2
Positive regulation of muscle cell differentiation	3/2
Negative regulation of insulin receptor signaling pathway	5/4
Positive regulation of phosphatidylinositol 3-kinsase activity	3/4
Insulin receptor signaling pathway	3/4

Mouse and human viruses target genes sharing similar cellular signaling pathways and GO terms despite large divergence in 3'UTRs. KEGG pathways and GO terms which were enriched for multiple miRNA and multiple virus strains in one species are listed.

### Viral miRNAs targeting specific host processes are potentially evolving under purifying selection

Many of the miRNAs encoded by EBV have been suggested to target host genes and were shown to be evolutionarily conserved [24]. Next, we investigated if the viral miRNAs that target specific host processes are evolving under differential pressure relative to other miRNAs. We obtained and aligned 14 CMV strain sequences [25] to reference the VirMir strain using ClustalW [26]. Given the multiple alignment we calculated the average conservation score for each miRNA, defined as a fraction of the consensus base at each position, averaged across the length of the miRNA. Those that putatively target specific host processes are significantly more conserved than the miRNAs that do not target a specific host pathway (Wilcoxon p-val 0.047), suggesting that they are evolving under purifying selection. This analysis could not be extended to other strains due to paucity of strains and inter-strain variability.

## Discussion

### Targeting specific host processes by viral miRNA are prevalent mechanisms of viral infectivity

Given that DNA viruses encode for miRNAs that can be effectively processed by the host machinery [27], it stands to reason that targeting of specific host genes by viral RNA may be effective ammunition against host defense. A single miRNA may target hundreds of genes for regulation, and identifying the true targets of miRNAs remains a challenge. This study was designed to investigate the possibility that virally-encoded miRNAs target host genes in pathways central to the persistence and/or replication strategies of many viruses. We first tested for enriched pathways among putative targets of known human viral miRNAs from miRbase. Recent next-generation sequencing efforts have revealed new viral miRNAs, and this trend is likely to continue as more cell types and tissues are analyzed [28] (and unpublished data by Trgovcich). Therefore, given that a vast majority of viral miRNAs remain to be identified, we used a dataset of predicted viral miRNA to test the generality of our findings for known miRNAs. Based on a stringent analysis, our results support the general hypothesis.

A few aspects of our finding are worth highlighting. First, published experimental data suggests that many of the pathways and processes putatively targeted by viral miRNAs make excellent targets to aid viral infectivity and survival. Second, we found that many KEGG pathways and GO biological processes are targeted by multiple miRNAs from multiple viruses, even across viral families. For instance, miRNAs targeting antigen processing and presentation and gonadotropin receptor hormone signaling are enriched in both adeno and herpes virus families. While it is known that herpes reside in neuronal cells during latency [29], axon guidance pathways are also enriched by both viral families, which is surprising because adeno viruses are normally associated with lung or gastrointestinal infections, and not neuronal cells [30]. The other commonality between both virus families was the cancer-related pathways, which we categorized as one group. Third, we also found many of these enriched targets in human to be the same when we repeated our analysis independently in mouse viruses. For example, cancer and the ErbB signaling pathway, insulin receptor regulation, and axon guidance pathways are preserved between mouse and human viral targets. We discuss a selection of enriched pathway in more detail below. Finally, we found evidence to suggest that the miRNAs targeting specific host processes are evolving at a relatively slower rate than other miRNAs. This is consistent with previous findings in EBV that showed viral miRNAs are likely to target host genes that are evolutionarily conserved [24]. We note that the evolutionarily conserved miRNAs in our analysis are not biased towards known miRNAs.

A few previous studies report similar findings, although in a limited context. A previous analysis showed enrichment for KEGG pathways associated with cell signaling and adhesion/junction pathways using the putative targets of 85 known miRNAs in 5 human herpes virus as an aggregate (not virus-specific or miRNA-specific) [19]. Another study of known KSHV miRNAs and targets predicted by differential expression analysis found the targets to be involved in proliferation, immune modulation, angiogenesis, and apoptosis [31]. Finally experimentally identified targets for EBV were found to be enriched for targets involved in innate immunity, cell survival, and cell proliferation [10]. Here we verified these previous finding and extended the analysis to individual viruses and miRNAs as well as broadened the virus families to include the adenoviruses and poxviruses. Furthermore, by including human and mouse viruses, our analyses support evolutionary prevalence of viral miRNAs targeting host pathways and processes for their survival and proliferation. Finally, we present evidence for possible purifying selection, acting on certain miRNAs targeting specific host processes.

### Caveats

While miRNA prediction is challenging in general, it is especially difficult to predict viral miRNAs, because most miRNA prediction approaches have been developed for eukaryotes and exploit evolutionary conservation [32], and are thus not applicable to viruses. Current miRNA prediction in viruses essentially relies on identifying hairpin like structures with certain additional properties [23]. We started with the putative miRNAs for dsDNA viruses compiled in the VirMir [23]. Undoubtedly, many of these predicted miRNAs will be false positives. However, the complete set of viral miRNAs is far from known. For instance, since 2007 in VirMir database, the number of known miRNAs for viruses studied here has grown greater than three-fold, and will grow as technologies improve and as additional cell types and tissues infected by viruses are analyzed. Using the group of all potential viral miRNAs based on predicted hairpin precursor structures therefore allowed us to begin with most comprehensive set of putative target genes for statistical analysis. Predicting miRNA targets presents yet another complication, especially for viruses, for similar reasons, and is fraught with false positives. A 24% false discovery rate has been calculated for MiRanda[33] similar to other target prediction algorithms. To minimize false positives from the putative miRNAs collected from VirMir, we restricted the target scores to a value of 140 or higher which correspond to exact seed matching criteria as well as a further threshold based on target score distribution for scrambled 3'UTR sequences. We adopted several additional checks to minimize false positives at the stage of functional enrichment assessment. First, we performed a strict pathway-specific adjustment of p-values accounting for 3' UTR lengths of genes in the pathway (a novel aspect of this work) and applied multiple testing corrections. Second, we used multiple virus families. Third, we relied on both KEGG and GO to better interpret our findings, and we used two distant species to corroborate our findings. Lastly, we compared the evolutionary conservation of miRNAs targeting specific host pathways to the other miRNAs based on 14 distinct strains of CMV and determined that they are conserved much more than expected, suggesting a selective pressure to maintain the targeting specificity. Although there are likely to be many false positives at the level of individual target genes, due to the reasons listed above, it is encouraging that they still reveal biologically meaningful patterns of functional enrichments.

### Pathways targeted by viral miRNAs

We found that miRNAs in many viruses targeted similar pathways even across distant host species. We compared the pathway enrichment for known viral miRNA in miRBase with that for predicted miRNAs in VirMir. For brevity, we discuss in detail the biological significance of three most significant pathways.

#### Cancer

It is encouraging that cancer, as a theme, is repeatedly revealed by our analysis. It is becoming increasingly clear that miRNAs may have a causal role in initiation and progression of cancer [34]. A few viruses included in this study are known to be associated with human cancer such as EBV in Burkett's lymphoma and nasopharyngeal carcinoma, and KSHV in Kaposi's sarcoma and some B cell lymphomas, but there is no direct evidence yet that viral miRNAs play a causal role in the development of cancer. Oncogenic pathways that viruses are known to interfere with include (1) cell growth, (2) proliferation, (3) evasion of apoptosis, (4) genetic instability, and (5) angiogenesis. The enriched KEGG pathways by both mice and humans include one pathway and three GO terms associated with features deregulated in cancer, in addition to the cancer pathway. Regulation of the MAPK signaling pathway is an essential co-factor for re-activation of KSHV [35]. A closely related PI3K pathway, targeted by herpes viruses, is critical to cell survival and growth [36], and is frequently activated in cancer pathways [37]. Both pathways have the potential to inhibit KSHV infections [38]. The PI3K pathway is a particular favorite for DNA viruses enabling infected cells to withstand viral induced stress [39]. DNA viruses can affect ErbB signaling pathway, which can initiate the MAPK pathway resulting in growth or differentiation [40]. Enrichment for these pathways suggests that viral miRNAs may play a role in the oncogenesis.

#### Hedgehog and insulin regulation

The Hh genes regulate tissue patterning and are expressed in the beta cells of the pancreas and was implicated in insulin production [41]. Hepatitis infection induces Hh pathways and is associated with fibrogenic repair, and vascular remodeling [42]. The viral need for tissue repair could be categorized as stress management so that the infected cell can withstand the stresses of viral replication programs; however, it is not clear why a virus would need to regulate insulin. Insulin is a metabolic molecule that causes tissue to take up glucose from the blood and to stop using fat as an energy source; it is possibly used as a metabolic regulator. One can speculate that viral hijacking of the key energy regulator may induce the infected cell to devote their energy production to the virus' needs. Enrichment of the GO term for negative regulation of insulin receptor signaling and insulin receptor signaling pathway would indicate that insulin regulation is indeed the targeted pathway.

#### Neuronal growth

HSV is commonly latent in nerves, and during an infection the virus travels along the nerves to the connected tissue and forms lytic lesions. Nerve growth factors are necessary survival factors for HSV in latency [43]. Additionally, it has been shown that HSV utilizes nerve growth cones and varicosities of axons for transport [44] indicating a role in nerve growth pathways. What is surprising is that three miRNA from two different adeno viruses were enriched for axon guidance pathway in humans. Because adeno viruses do not usually target neurons, it is likely that this is a false positive. Nevertheless, viral miRNA targets are enriched for axon guidance and GO term neuron development and support the targeting of this important latency pathway for the herpes virus.

## Materials and methods

### MiRNA target prediction

Given a miRNA sequence from VirMir [23], and the 3' UTR of human (or mouse) genes, we used Miranda3.3 to predict the miRNA targets [45]. As a baseline threshold, we only considered Miranda hits scoring 140 or higher, which corresponds to a perfect heptamer match in positions 2-8 and should be considered stringent [45]. As a further control, we randomly scrambled each 3' UTR sequence and repeated the target prediction on the randomized sequences. Using the distribution of scores on the randomized sequences, we determined a score cutoff corresponding to top 1% (for analysis of known miRNA) or 5% (for analysis of predicted miRNAs) scores. This threshold was determined separately for each miRNA and was always much more stringent that the baseline score threshold of 140.

### Functional enrichment

We compared each gene target set with an annotated functional gene sets corresponding to KEGG pathways and GO biological processes; gene sets with fewer than 10 members were excluded. For each miRNA we assessed overlap between the miRNA target set and each functional gene set using a hypergeometric test, which estimates the probability (p-value) of observing an overlap of certain size of greater between two gene sets by random chance. We noted that if the genes in functional gene set have longer 3' UTRs, then those pathways are more likely to be deemed as enriched simply by chance. To control for 3' UTR length bias, we divided the range of 3' UTR lengths into 20 equal-sized bins. For a miRNA, for each target of that miRNA, we randomly selected a gene in the same 20 percentile bin, thus generating a length matched random set of target genes for each miRNA; we generated 10 such random sets corresponding to each actual target set. For each randomize target set, we calculated the hypergeometric p-value for the overlap with each functional gene set. Thus, for a functional gene set (KEGG or GO), we generated a NULL distribution of hypergeometric p-values containing 68,090 p-values corresponding to 10 randomized sets corresponding to 6809 miRNAs. Given an actual miRNA target set *S *and functional gene set *F*, and given the hypergeometric p-value *P(S, F)*, we estimated an adjusted p-value *P^Adj^*(S, F) as the rank of *P *in the 68,090 p-values corresponding to *F*. Finally, given adjusted p-values for all miRNA target sets for all viruses against all functional gene sets, we corrected the adjusted p-values for multiple testing using the Benjamini-Hochberg procedure for FDR estimation [46].

## Competing interests

The authors declare that there are no competing interests

## Authors' contributions

S.H. and J.W.C. conceived and designed the project. J.W.C. performed the analyses. J.T. provided the virology expertise in selecting some of the datasets used as well as the discussion. S.H. and J.W.C. wrote the manuscript with critical help from J.T.

## Additional files

Predicted miRNA targets (310 unique Human miRNA, 817 unique Entrez gene ID's) are available at ftp://ftp.cbcb.umd.edu/pub/data/sridhar/Human.miRNA.supplemental.

## Declarations

The publication costs for this article were funded by NIH grant R01GM085226 to SH.

This article has been published as part of *BMC Bioinformatics *Volume 14 Supplement 2, 2013: Selected articles from the Eleventh Asia Pacific Bioinformatics Conference (APBC 2013): Bioinformatics. The full contents of the supplement are available online at http://www.biomedcentral.com/bmcbioinformatics/supplements/14/S2.
